# Validation of direct CT measurement of malrotation in femoral neck fractures: A bone model study

**DOI:** 10.1371/journal.pone.0278850

**Published:** 2023-04-04

**Authors:** Emmanouil Liodakis, Gesa Helen Pöhler, Lena Sonnow, Philipp Mommsen, Jan-Dierk Clausen, Tilman Graulich, Alexander Maslaris, Mohamed Omar, Timo Stübig, Stephan Sehmisch, Tarek Omar Pacha

**Affiliations:** 1 Trauma Department, Hannover Medical School (MHH), Lower Saxony, Germany; 2 Department of Radiology, Hannover Medical School (MHH), Lower Saxony, Germany; 3 Department of Orthopaedics and Trauma Surgery, Alfried Krupp Hospital, Campus Rüttenscheid, Essen, Germany; University Hospital Zurich, SWITZERLAND

## Abstract

**Background:**

While postoperative malrotation in the subtrochanteric region is a well-known problem, malrotation after osteosynthesis in proximal femoral fractures has not been extensively studied. In this context, many methods for perioperatively assessment of femoral torsion have been described, but none of them is applicable in the basicervical region of the proximal femur. As an important difference in femoral neck fractures, the discontinuous neck fails to serve as a significant “pointer” for measurements and malfunctions to be placed in relation to the condylar plane. Considering postoperative maltorsion at any location as a substantial negative effect on patients’ outcome and functional expectations, precise and patient-friendly rotation measurement standards in femoral neck fractures are desired in clinical practice. Recently, a novel computed tomography (CT) based geometric technique was described named “direct measurement” with promising results covering this diagnostic disparity, but still requires validation. Thus, we aimed to validate the previously described technique using a controlled range of displacement in a femoral neck fracture Sawbone^®^ model.

**Methods and findings:**

A goniometer was designed to set retro- and anteversion of the proximal femur in a reproducible manner. Prospectively, all femurs underwent a CT scan and were measured 3D for displacement. The interclass correlation between the CT measurements and the goniometer measurements was calculated and was found to be very high (1.00, 95% confidence interval: 0.99–1.00; p < 0.001). For the mean of all measurements, the Pearson’s correlation was 1.00 (p < 0.001). No significant differences in the measurements of both investigators were observed, with 20° of retroversion not significant (-1.20 ± 1.71; 95% confidence interval: -2.43–0.03; p = 0.054).

**Conclusion:**

This CT-based 3D measurement technique may allow for perioperative malrotation assessment in basicervical femoral neck fractures and appears to be feasible in femoral neck fractures when it comes to rare cases of osteosynthesis. Further investigations are still needed to define the thresholds of malrotation provoking functional impairment after osteosynthesis in basicervical femoral neck fractures.

## Introduction

Proximal femoral fractures are ranked third of all fracture types [1, 2], hospitalizing approximately 300,000 patients per year in the United States, with an increasing tendency and estimated range of 6–21.3 million patients worldwide by 2050 [3, 4]. Proximal femoral fractures can be stratified by location into: subtrochanteric, intertrochanteric, and femoral neck fractures (FNFs) [4]. FNFs are gaining more importance, being a typical fracture of the elderly patient, with an incidence of 100,000 per year in the German population and affecting about 30% of all women [5]. Regarding FNFs, osteosynthesis, total hip arthroplasty, and hemiarthroplasty have to be considered, depending on the fracture type, patient age, and compliance [4, 6]. Valgus impacted and less-displaced fractures (Garden type I and II) can be treated by osteosynthesis, even in elderly patients [4, 5, 7]. Patients with higher grades of displacement are treated by hemiarthroplasty or total hip arthroplasty in older patients and by osteosynthesis in young patients [4, 6, 7].

In FNFs, subcapital and midcervical fracture types are common, and are mostly treated by arthroplasty, especially in elderly patients. In contrast, basicervical FNFs (1.8–7.6% of all PFFs) [4, 8] and higher displaced fractures in the midcervical area in young patients are treated with cephalomedullary nails, dynamic hip screws, or cancellous screws [4, 5, 8, 9]; thus, the best method is still under discussion [10]. While postoperative malrotation in the subtrochanteric region is a common and well-known problem [11–13], malrotation after osteosynthesis in FNFs, especially in the basicervical region, is less often described [7, 14]. Also, a generally accepted threshold from which one has to perform corrective therapy is still missing.

Given the widely accepted practice of providing joint replacement in elderly, this fact may be one explanation for the only few literatures of malrotation after osteosynthesis in patients with fractured femoral necks. The postoperative diagnosis of malrotation in adults is typically a domain of computed tomography (CT) [15, 16], engaging various geometrically based measurement techniques [16–18]. All measurement techniques of femoral shaft malrotation use the femoral neck as a “pointer,” assessing ante- or retroversion compared to the distal femoral joint line. In fractures proximal to the intertrochanteric line, this “pointer function” cannot be applied as the neck is fractured. Therefore, in 2021, a simple geometric and CT based measurement technique was introduced for the assessment of malrotation of fractures proximal to the intertrochanteric line to precisely define the torsional error remaining postprocedural after osteosynthesis. In detail, the radiological measurement is performed in two sectional images and the angle of malrotation is calculated “directly” without reference needed to the condyles. Despite promising results of the previous study, the proof of validity of this new technique remained to be determined [19]. Thus, we aimed to validate this approach using a controlled range of displacement in FNFs in a Sawbone^®^ model.

## Materials and methods

### Femoral neck goniometer

**Fig 1:**
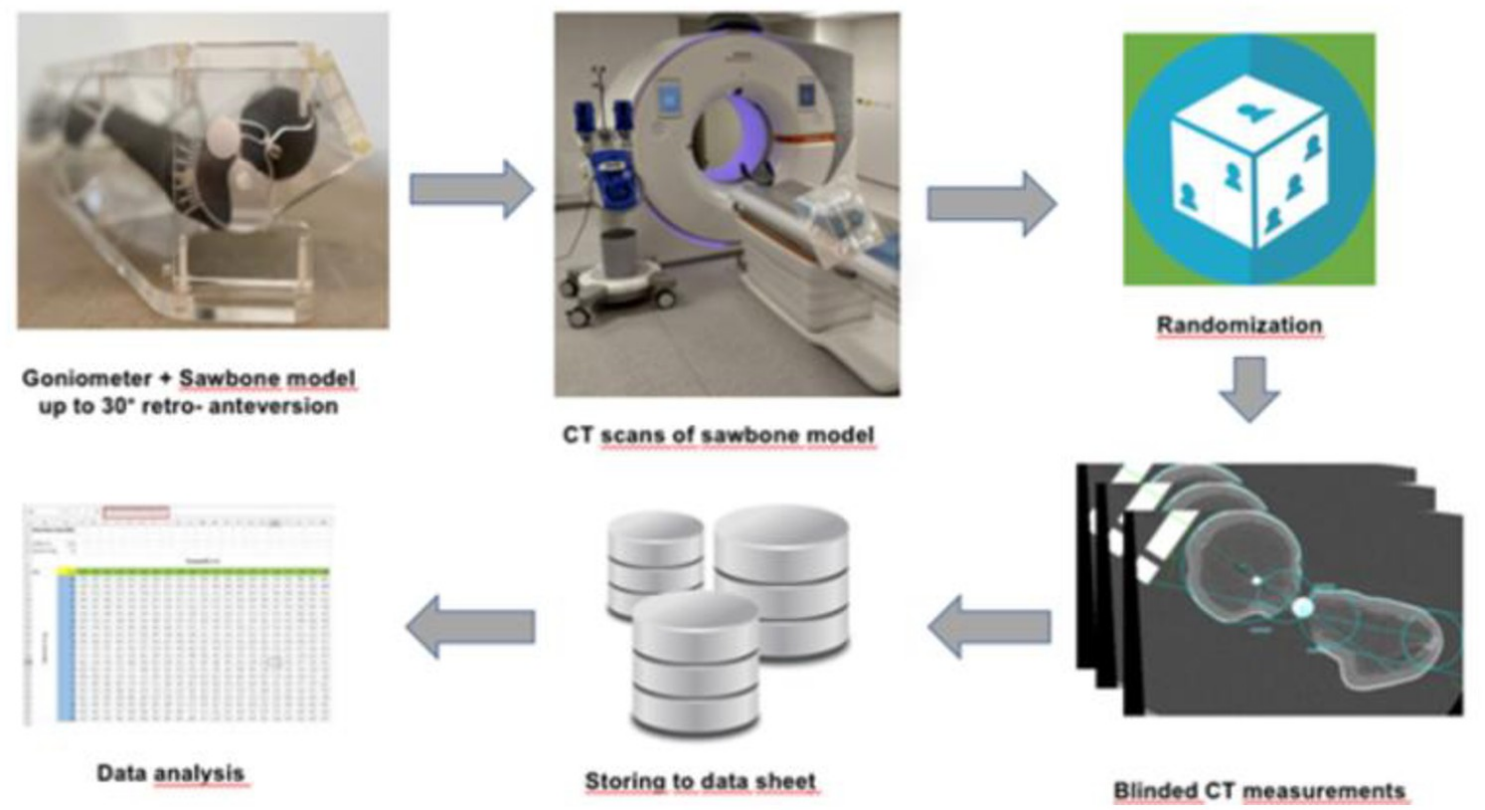
A graphical overview of the study design is shown.

**Fig 2:**
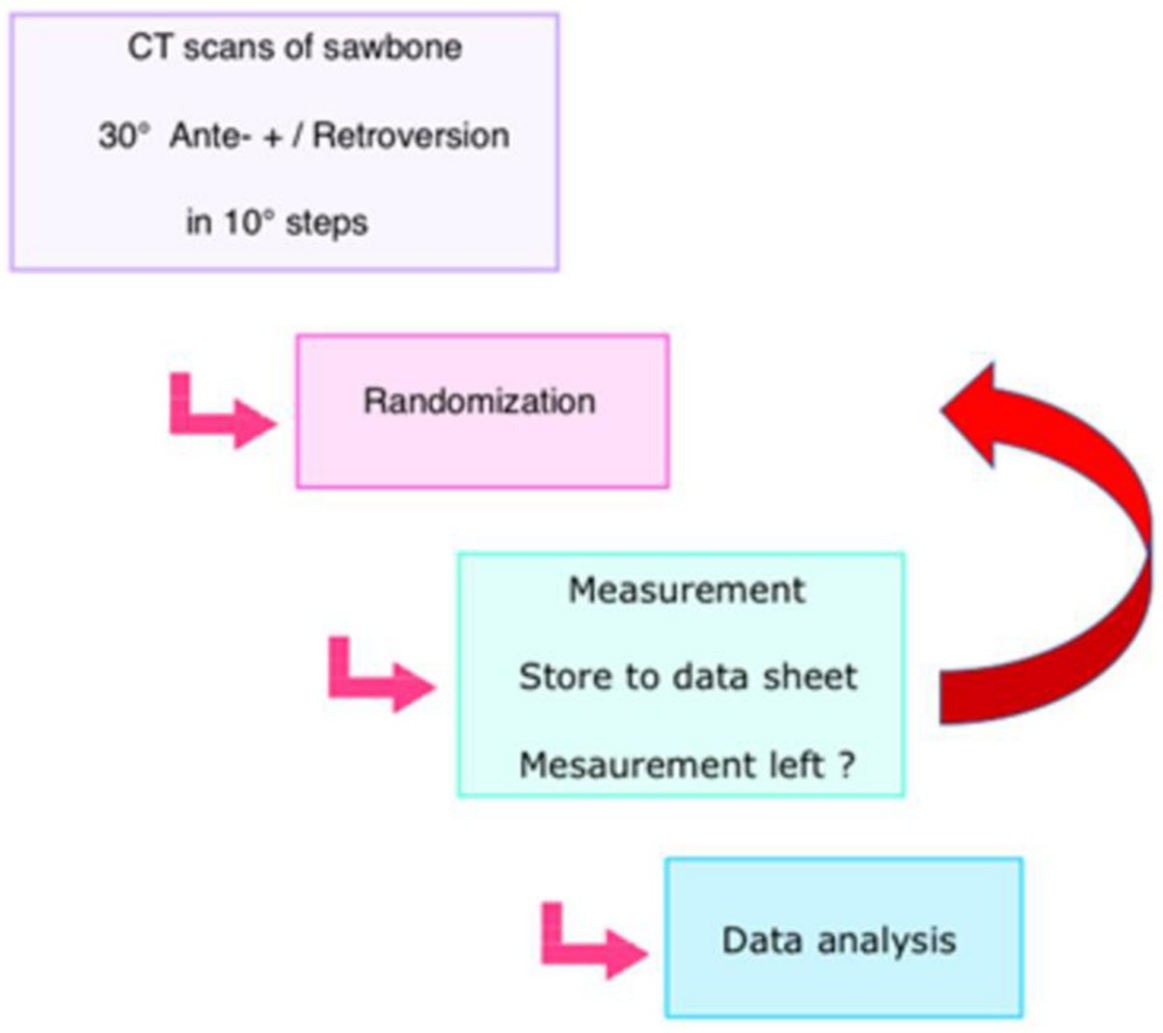
Workflow. The roadmap of the validation study is demonstrated.

We designed a goniometer to set retro- and anteversion of the proximal femur in a precise and reproducible manner (Figs 1 and 2). A femoral neck osteotomy was performed at the basicervical region of a radiopaque left femoral Sawbone^®^ (Sawbones Europe AB, Malmö, Sweden) and the femur was placed into the device. Fig 1A shows a view from above, presenting the angulation axis. The displacement axis was slightly angulated in the frontal view but centered axially (Fig 1B). On the goniometer there is a scale indicating the degree of dislocation, which can be set in 10° increments (2A). As shown in Fig 2B and 2C, the femoral neck can be angulated along this axis in a range from 30° retroversion to 30° anteversion. The device is made of plastic to avoid influence of the radiopaque structures, and the Sawbone^®^ is attached by commercially available small fragment cortical screws.

The investigation was carried out on fully anonymized Sawbone^®^ data. Approval of the local ethics review board was obtained for our study (Ethics Committee of Hannover Medical School). An assessment by the ethics committee and the in-house data protection officer is available. No concerns were raised. Informed consent was obtained in writing language.

### CT measurements

The original measurement technique described in 2021 [19] was modified to accurately define the torsional error remaining periprocedural in FNFs.

**Fig 3:**
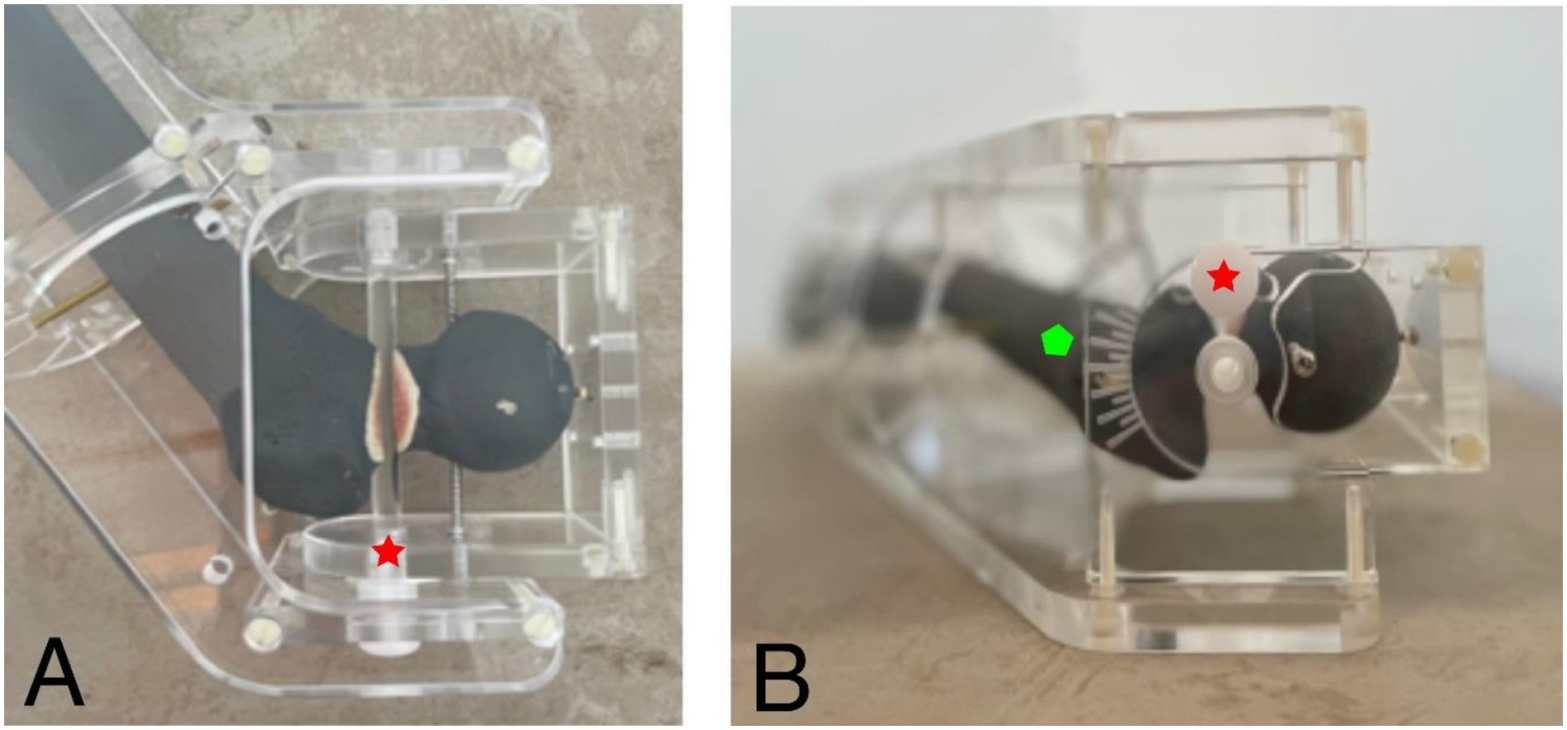
Overview of the goniometer device including the fixed Sawbone^®^ model. **A** rotation axis of the fracture line (red star). **B** axial view of the device. The green pentagon marks the displacement scale. The red star depicts the rotational axis.

**Fig 4:**
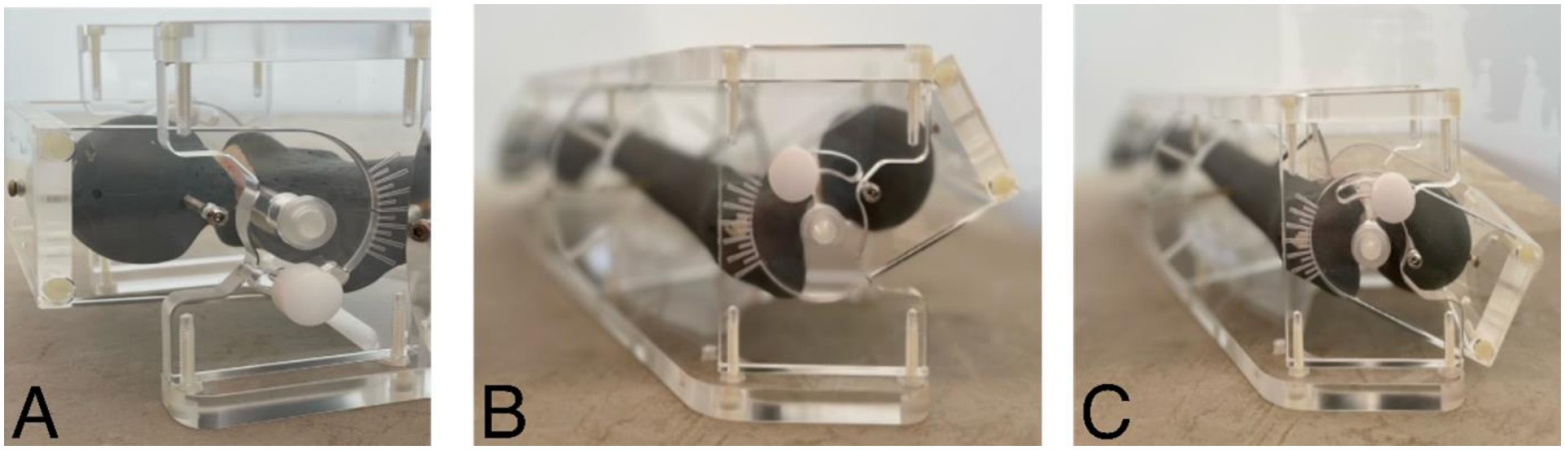
Overview of the sawbone^®^ fixed in the goniometer and the measurement presets. **A** view of scale with 10° increments. **B** malrotation set to 30° anteversion. **C** malrotation set to 30° retroversion.

In detail, plane sections of 3D CT imaging of the proximal femur were aligned in all 3 planes and centered along the femoral neck using a dedicated imaging-software (Visage^®^, Visage Imaging, Berlin, Germany) (Fig 3A–3C). In frontal view the section is guided down near the Adam’s bow. CT slices of 5 mm thickness were acquired through the femoral neck region. In axial sectioning a tangent is drawn along the frontal cortex of the greater trochanter (Fig 4A). The section should not be changed from this point on. Next using the medical-planning software MediCAD (mediCAD Hectec GmbH, Landshut, Germany), a parallel line to the tangent is drawn at the dorsal border of the femoral neck, crossing the lesser trochanter (Fig 4B). A best fitting circle is placed centered in the femoral head and additionally centered in the lateral opening of the femoral head shown in Fig 4C. Next two circles are fit between the tangent and the parallel line (Fig 4D). The connecting line between the center points of both, the medial circles in the femoral head and the lateral circles in the femoral neck respectively mark the central axis line of the medial and the lateral femoral neck (Fig 4E). The intersection points of both axes allow the measurement of the real angulation of the femoral neck and proximal femoral fracture (Fig 4E).

### CT data evaluation

**Fig 5:**
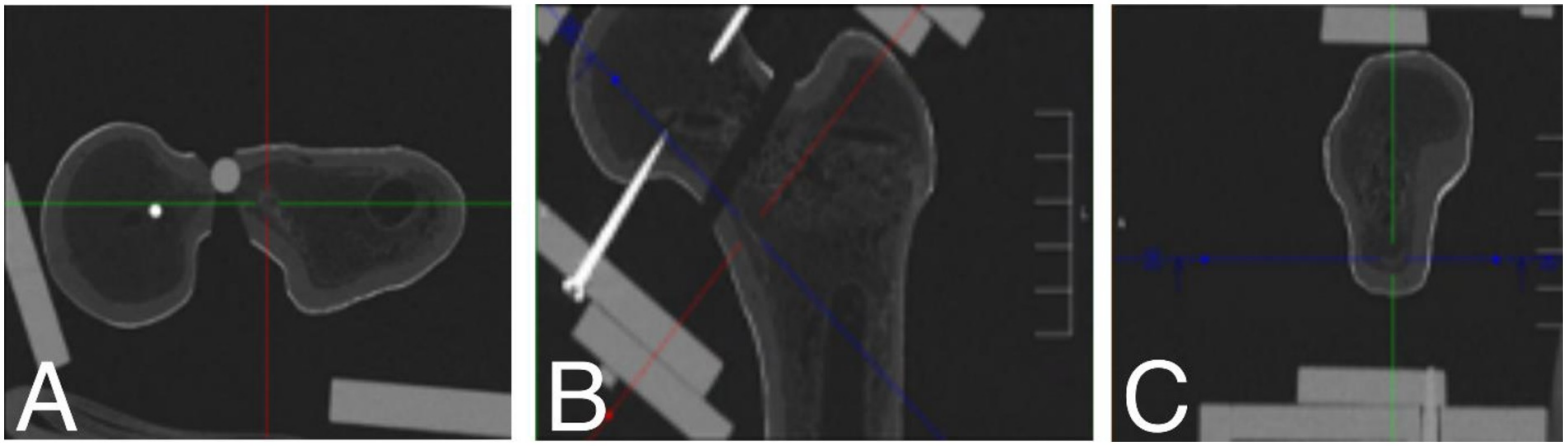
An exemplary 3D CT scan of the synthetic femoral bone model fixed by means of the goniometer is shown. The alignment of section planes in all three dimensions is depicted: **A** axial, **B** coronal and **C** sagittal view using Visage^*®*^.

**Fig 6:**
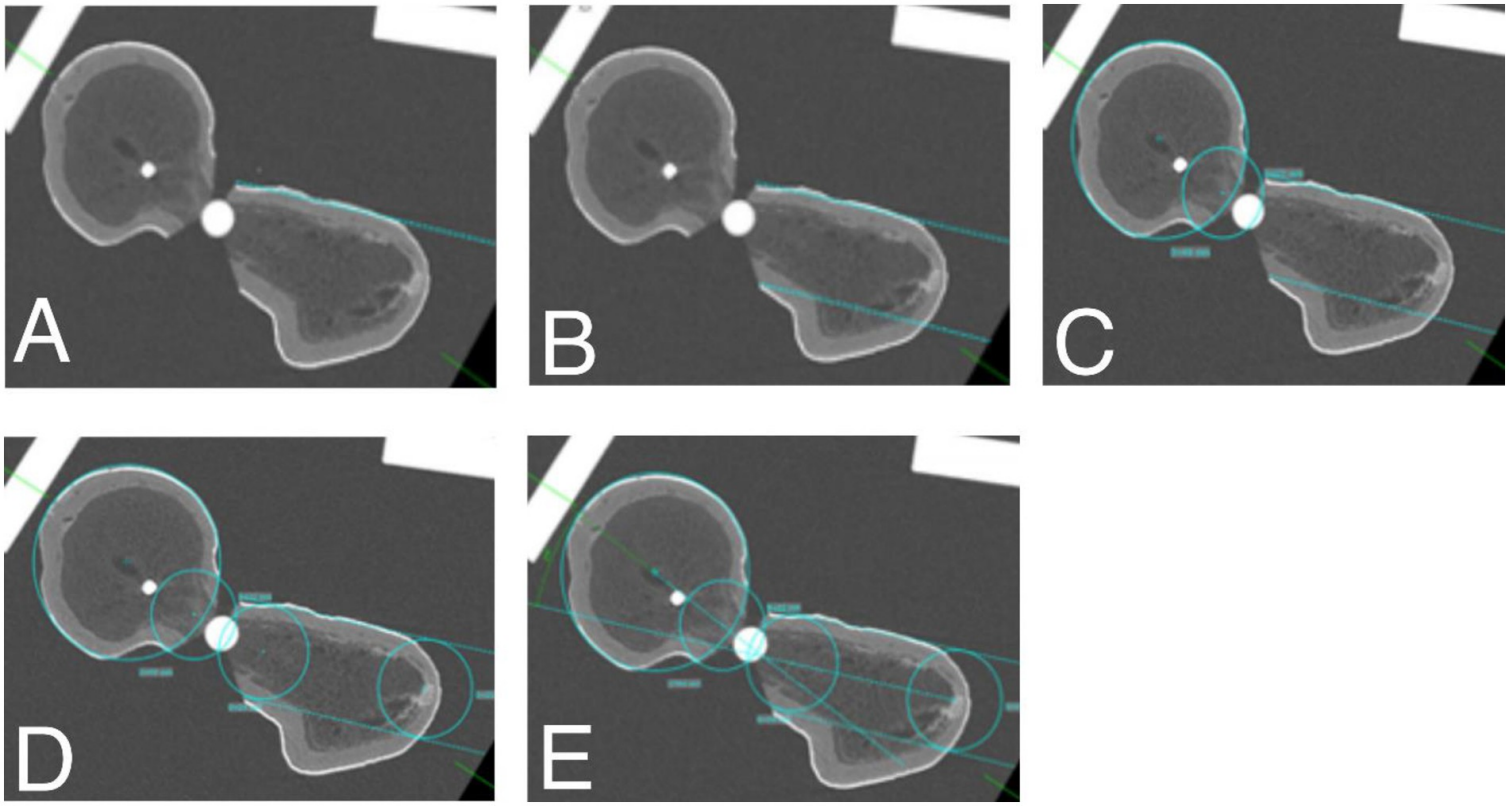
**A** axial plane with tangent to the ventral cortex of the greater trochanter. **B** axial section with parallel line to the tangent crossing the lesser trochanter. **C** axial plane with best-fitting circle in the femoral head and lateral head opening. **D** axial plane with best-fitting circles defining the lateral femoral neck. **E** transverse section showing the crossing point of both circles connecting axis.

The workflow of the study is visualized in Figs 5 and 6. Two investigators performed the measurements for each defined degree of deformity. Both investigators are board certified, one in orthopedic trauma and the other in radiology. The clinical experience of each investigator is longer than 6 years. All measurements were performed on "Visage 7.1"(Visage Imaging, Berlin, Germany) and mediCAD (mediCAD Hectec GmbH, Landshut, Germany) running on a personal computer (Windows 10 Professional Microsoft Corporation, One Microsoft Way, Redmond, WA USA).

First the Sawbone was attached to the femoral neck goniometer and a CT scan was performed in 10° steps from 30° of anteversion to 30° of retroversion. The scans were stored and blinded. Each investigator took randomly the first scan, applying the measurement technique described above. The result was stored in a data sheet and the next scan was taken randomly to perform the next measurement. While performing the measurements, the real degree of displacement was undisclosed to the investigators. This procedure was repeated by each investigator until each degree of displacement was measured ten times by both investigators. Next the statistical analysis was performed.

Since there was no patient recruited, and no personal data collected, no concerns were raised neither by the local ethical committee or the in-house data protection officer.

### Statistical analysis

The statistical analysis was performed using SPSS (SPSS 28.0, IBM Corp., Armonk, NY, USA) using a commercial personal computer (Windows 10 Professional Microsoft Corporation, One Microsoft Way, Redmond, WA USA). Descriptive analysis was done defining minimum, maximum, mean, standard variations and variances. The interclass correlation (ICC) was calculated to define the interrater reliability and the Pearson’s score for correlation values. Two-way mixed model and absolute agreement was used. The quality of reliability was considered as poor with ICC < 0.5 and on the other hand as good/excellent with ICC > 0.8–0.9 as described by Fleiss et al. [20] Paired Student’s t-test are performed for comparison of both investigators for each degree level (30-degree retroversion to 30-degree anteversion).

The level of significance was set by alpha = 0.05 (two tailed) for ICC and paired Student`s t-test respectively. For the Pearson’s correlation alpha was set to 0.01 (two tailed).

## Results

### Displacement measurements

A descriptive analysis of the measurements for each degree level was performed. Each investigator performed ten measurements for each displacement level (30-degree anteversion to 30-degree retroversion in 10-degree steps) (Table 1).

**Table 1:** Descriptive statistics showing minimum, maximum, mean, standard deviation and variance for each step of displacement.

	Number	Min (°)	Max (°)	Mean (°)	Standard deviation (°)	Variance (°)
**Inv1_10°_ante**	10	9.60	12.40	11.00	0.89	0.80
**Inv2_10°_ante**	10	10.30	12.00	11.19	0.56	0.32
**Inv1_20°_ante**	10	17.60	23.90	20.05	1.89	3.61
**Inv2_20°_ante**	10	17.50	21.30	19.80	1.18	1.36
**Inv1_30°_ante**	10	28.10	35.10	30.72	2.32	5.40
**Inv2_30°_ante**	10	28.90	31.80	30.20	1.08	1.16
**Inv1_10°_retro**	10	5.40	11.10	8.20	1.83	3.34
**Inv2_10°_retro**	10	7.50	10.90	9.11	0.99	0.98
**Inv1_20°_retro**	10	16.20	19.90	17.78	1.26	1.58
**Inv2_20°_retro**	10	17.90	20.70	18.98	0.99	0.99
**Inv1_30°_retro**	10	26.80	35.40	29.43	2.49	6.21
**Inv2_30°_retro**	10	27.90	31.20	29.67	1.08	1.17

### Correlation analysis

The inter class correlation (ICC) for all ten measurements and both investigators are shown in Table 2. The mean ICC with 0.999 (95%CI: 0.99–1.000; p<0.001) shows a high objectivity of the method. Taking a closer look at the single measurement the lowest value for the ICC is seen in investigation 1 (ICC = 0.96 95%CI: 0.72–0.99; p = 0.001) and highest in Investigation 10 (ICC = 1.00 95%CI: 0.99–1.00; p < .001) (Table 2). For each measurement the Pearson’s correlation was calculated, see Table 2.

**Table 2:** ICC calculation and Pearson’s score for both investigators for each of the ten investigations.

Investigation number	ICC	95% CI		Pearson‘s	p-value	
		lower	upper		ICC	Pearson‘s
**Mean (all)**	0.99	0.99	1.00	0.99	<0.001	<0.001
**No.1**	0.96	0.72	0.99	0.97	0.001	0.001
**No. 2**	0.98	0.85	0.99	0.96	<0.001	0.002
**No. 3**	0.98	0.89	0.99	0.96	<0.001	0.002
**No. 4**	0.99	0.97	1.0	0.99	<0.001	<0.001
**No.5**	1.00	0.86	1.00	0.99	<0.001	<0.001
**No. 6**	0.99	0.84	1.00	1.00	<0.001	<0.001
**No. 7**	0.99	0.96	1.00	0.99	<0.001	<0.001
**No. 8**	0.98	0.87	1.00	0.96	<0.001	0.002
**No. 9**	0.99	0.97	1.00	0.99	<0.001	<0.001
**No. 10**	0.99	0.99	1.00	1.00	<0.001	<0.001

For the mean of all measurements, the Pearson’s correlation was 1.00 (p<0.001). The lowest value is performed in investigation 1 (0.97, p<0.001),) with the highest values for investigation 4 and 10 with both 1.00 (p<0.001) (Table 2).

### Comparison of displacement measurements

For each degree level from 30° anteversion to 30°retroversion, the significance of the mean differences of the measurements of the investigators was calculated by paired Student’ t-test. The results seen in Table 3 show no significant differences in the measurements of both investigators with 20° retroversion being almost not significant (-1.20 ± 1.71/ 95%CI: -2.43–0.026; p = 0.054).

**Table 3:** Overview of paired Student’s t-test for each step of displacement.

		Mean (°)	Standard deviation (°)	95% CI		p-value
				lower	upper	
pair 1	10° ante	-0.19	0.69	-0.69	0.31	0.409
pair 2	20° ante	0.25	1.94	-1.14	1.64	0.694
pair 3	30° ante	0.52	2.59	-1.33	2.37	0.541
pair 4	10° retro	-.91	2.10	-2.41	0.59	0.204
pair 5	20° retro	-1.2	1.71	-2.43	0.03	0.054
pair 6	30° retro	-.24	3.24	-2.56	2.08	0.820

## Discussion

This study validates a recently described CT-based measurement technique to assess the malrotation in basicervical FNFs. The resulted very high mean ICC for both investigators confirms a high-ranking objectivity of the presented method. Despite the excellent values of the pearson correlation analysis and ICC, a slice effect of learning can be seen in the data. The values for ICC and the values for Pearson’s score increase from investigation 1 with the lowest values to investigation 10 with the highest values. The results showed no significant differences in the measurements of both investigators.

The assessment of femoral malrotation has a long history and many different techniques were proposed to assess the malrotation geometrically in computed scans with a threshold defined for correction in femoral shaft and intertrochanteric fractures above 15° [11, 13, 17, 21]. The most common CT-based techniques are those reported by Jarrett et al. and Kaiser et al. [16–18]. As a limitation, these methods use the femoral neck as a “pointer” relative to the distal femoral joint line. Therefore, the femoral neck must be untouched. When it comes to fractures proximal to the intertrochanteric line including basicervical FNFs, these standard methods no longer work, as the femoral neck is affected [16–19]. Considering especially these patients, our presented method might enable a standardized measurement approach in patients with basicervical FNF´s undergoing a CT scan.

In general, any malrotation of the femur comes with impairment in patient outcomes, regardless of location, when considering osteosynthesis. While issues with malrotation in the femoral shaft are described [11, 12, 17, 21–25], Maléř et al. report a doubling of cutout rate when malrotation exceeds 15° in the intertrochanteric region, showing the need for anatomic reduction [26]. Fractures of the basicervical femoral neck are treated with osteosynthesis. So, it can be assumed, that malrotation also occurs and probably leads to similar issues comparable to intertrochanteric and femoral shaft fractures. Interestingly, there are no data reported regarding this issue in the literature. Consequently, no threshold for correction has been defined regarding basicervical FNFs. In FNFs, a posterior tilt exceeding the threshold of 20° in Garden type I and II fractures increases the failure rate of osteosyntheses fourfold [27]. Kalsbeek et al. report a rate of treatment failure for FNF Garden type I and II with posterior tilt over 20° being similar to displaced FNFs (Garden type III/IV) [27]. Therefore, a proper reposition in axial view is necessary. A preoperative and precise assessment of the posterior tilt is optimal for the surgeon to determine the need for optimal reduction. Taking into account that this subtype of Garden type I and II with posterior tilt greater than 20° behaves like an unstable fracture, causing avascular necrosis due to vascular kinking and tearing, an exact preoperative assessment, especially in elderly patients, may elicit primary arthroplasty [24, 25]. However, because usually applied measurement techniques do not fit for areas proximal to the intertrochanteric line, an objective and valid technique to measure the amount of malrotation in this area seems necessary for clinical translation.

From a clinical point of view, the presented CT-based method might be effective to preoperatively assess malrotation proximal to the intertrochanteric line, as well as postoperatively regardless of persisting osteosynthesis. As a matter of fact, the data provided here on the Sawbone^®^ model, show reliable results regarding ICC and Pearson’s score in displaced basicervical FNFs without osteosynthesis. Nevertheless, in the proof of concept study published in 2021, the measurements were conducted on postoperatively collected CT scans including osteosynthesis with comparably good results [19]. This reflects that osteosynthesis does not substantially affects our technique. Therefore, this method adds to other, well described, techniques [16–18], as it is designed for special cases like osteosynthesis in basicervical femoral neck fractures and rare cases of FNFs intended to be treated with osteosynthesis.

Regarding validation, for the test set-up, we chose a bone model using a sawbone instead of an experimental set up with cadaver. Sawbone material provides different advantages. It is stable in shape, density and size and can be flexible modelled. In comparison to cadaver studies, special storage techniques do not have to be considered and costs are lower attaining the same level of test set-up comparability and reproducibility. Furthermore, our synthetic bone model study built on the previously described real bone study yields results comparable to a two-phase test set-up [28].

There are some limitations of this study. First, the method is only applicable if a CT scan is available. As unnecessary radiation must be avoided, a CT scan is performed only if a clinically relevant malrotation (>15°) is suspected or if increasing pain or impairment in mobility is clinically detected. In these cases, postoperative malrotation and other entities like nonunion or implant failure need to be ruled out, making additional radiation reasonable [16]. Additionally, in patients of clinically indicated CT´s, the mean volumetric CT dose index and the mean dose-length product might be decreased based on the “as low as reasonable “concept, scanning only the precisely defined anatomical area of the femur, increasing pitch or noise index and reducing tube voltage while preserving imaging quality. In contrast to established techniques of femoral shaft malrotation measurements, our approach even decreases radiation exposure as the condyles are not irradiated. This decreased radiation exposure per patient may also enable the possibility for an intraoperative monitoring application. Second, the intervals of displacement were set at 10° in a range from 30° retroversion to 30° anteversion. In a future study, a smaller interval of displacement such as 5° intervals may be preferable as 10° might be clinically rather significant. However, the maximal standard deviation in our study was seen in 30° retroversion for investigator 1 and in 20° anteversion for investigator 2. The maximal variance for the measurements also differed between both specialists. Thus, a defined 5° increment would not have covered all resulted variances in this validation study with some cases exceeded the 5° mark. Third, the measurements were performed by two experienced clinicians, specializing in orthopedic trauma and radiology, performing ten measurements for each degree of displacement. This manageable number for each degree of displacement needs to be taken in account when interpreting the data provided in this study. To counter this, a highly controlled setting was chosen, making the scan results distinctly reproducible.

In contrast to femoral shaft fractures, incidences of malrotation, tolerable limits and indications for surgical correction have not been precisely defined for proximal femoral fractures [15, 17]. However, since femoral shaft malrotation decreases quality of life and increases functional impairment, more research is needed to correlate malrotation in basicervical FNFs with patient’s clinical outcome, too.

## Conclusion

In this study, a simplified method to measure femoral neck rotation in CT scans perioperatively has been validated using a sawbone model as an important further step in its clinical translation from bench to bedside. The results indicate that this method may be helpful assessing perioperatively malrotation in basicervical FNFs and appears to be feasible in FNFs, when it comes to the rare case of osteosynthesis. Further investigations are still needed, to define thresholds of malrotation provoking functional impairment after osteosynthesis in basicervical FNFs.

## Supporting information

S1 File (XLSX)

S2 File (XLSX)
